# Incretin Receptor Null Mice Reveal Key Role of GLP-1 but Not GIP in Pancreatic Beta Cell Adaptation to Pregnancy

**DOI:** 10.1371/journal.pone.0096863

**Published:** 2014-06-13

**Authors:** R. Charlotte Moffett, Srividya Vasu, Bernard Thorens, Daniel J. Drucker, Peter R. Flatt

**Affiliations:** 1 SAAD centre for Pharmacy and Diabetes, University of Ulster, Cromore Road, Coleraine, Northern Ireland; 2 Centre for Integrative Genomics, University of Lausanne, Lausanne, Switzerland; 3 The Lunenfield – Tanenbaum Research Institute, Mt. Sinai Hospital, University of Toronto, Toronto, Ontario, Canada; University of Lancaster, United Kingdom

## Abstract

Islet adaptations to pregnancy were explored in C57BL6/J mice lacking functional receptors for glucagon-like peptide 1 (GLP-1) and gastric inhibitory polypeptide (GIP). Pregnant wild type mice and GIPRKO mice exhibited marked increases in islet and beta cell area, numbers of medium/large sized islets, with positive effects on Ki67/Tunel ratio favouring beta cell growth and enhanced pancreatic insulin content. Alpha cell area and glucagon content were unchanged but prohormone convertases PC2 and PC1/3 together with significant amounts of GLP-1 and GIP were detected in alpha cells. Knockout of GLP-1R abolished these islet adaptations and paradoxically decreased pancreatic insulin, GLP-1 and GIP. This was associated with abolition of normal pregnancy-induced increases in plasma GIP, L-cell numbers, and intestinal GIP and GLP-1 stores. These data indicate that GLP-1 but not GIP is a key mediator of beta cell mass expansion and related adaptations in pregnancy, triggered in part by generation of intra-islet GLP-1.

## Introduction

GLP-1 and GIP are incretin hormones released from intestinal enteroendocrine cells in response to feeding [1]–[3]. In addition to glucose-dependent stimulation of insulin secretion, they exert a variety of other actions on beta cells including stimulation of insulin biosynthesis and beta cell replication together with protection against chemical attack and inhibition of apoptosis [1]–[4]. Other actions of GLP-1 include inhibition of glucagon secretion, gastric emptying and feeding, with additional positive effects on cardiac muscle and, in common with GIP, improvement of cognition and bone formation [5]–[8]. These attributes of GLP-1 have been captured for treatment of type 2 diabetes by development of stable GLP-1 mimetics and DPPIV inhibitors which inhibit the normal rapid degradation of both incretin hormones [9]–[11].

Much has been elucidated concerning the pancreatic and extrapancreatic actions of GLP-1 and GIP together with mechanisms regulating the secretion of the two incretin hormones from intestinal L- and K-cells, respectively [4], [12]–[17]. However, recent studies have opened a whole new aspect of research by demonstrating that GLP-1 and GIP are not generated exclusively in the gut but may also be present in islet cells. Thus, recent studies have shown that the normal proglucagon processing to glucagon in islet alpha cells by PC2 can be modified by expression of PC1/3 yielding GLP-1 and related peptides normally produced by intestinal L-cells [18]–[27]. Accordingly GLP-1 has been demonstrated by immunochemical staining, immunoassay, bioassay and mass spectroscopy techniques in both animal and human alpha cells, giving rise to speculation that islet-derived GLP-1 may play a key role in beta cell function. Use of antibodies or chemical antagonists of GLP-1 indicate that GLP-1 released from islet alpha cells *in vitro* may stimulate insulin release from adjacent beta cells via paracrine or local islet cell interactions [24], [26]. Further studies also indicate that GIP (1–42), or more likely the equally biologically active fragment GIP (1–30) generated by the action of PC2, is also produced by islet alpha cells [28]. More recently still, transgenic mice with global deficiency in proglucagon-derived peptides have been shown to exhibit ectopic expression of biologically active GIP in islet beta cells [29]. Taken together, these observations suggest that GLP-1 and GIP are generated within islets and exert possible unsuspected roles in the functional regulation of beta cells and other islet cell types.

Some evidence exists for physiological significance of islet-derived GLP-1 and GIP in terms of insulin secretion [24], [26] but their involvement in the regulation of beta cell mass is possibly more intriguing given the paucity of agents with such effects and the loss of beta cells in both type 1 and type 2 diabetes [30]–[32]. Pregnancy is one of the very few situations associated with physiological and reversible expansion of beta cell mass not only in animals, which show remarkable plasticity of insulin secreting cells, but also in humans [33]–[38]. Given the positive actions of the two incretins on beta cell mass, resulting from reciprocal effects on beta cell proliferation and death [1], [2], we examined the role GLP-1 and GIP in islet adaptation to pregnancy using incretin receptor knockout mice [39]–[41]. The results reveal an important role of GLP-1 in pregnancy-induced increases in beta cell mass, mediated largely by local GLP-1 production in alpha cells. In contrast, GIPR KO mice demonstrated intact mechanisms of islet adaptation to pregnancy, suggesting that islet or K-cell derived GIP is not essential for pregnancy-associated expansion of beta cell mass.

## Methods

### Animals

Adult 8-week-old female C57BL/6 mice, GLP-1RKO mice and GIPRKO mice (n  =  6) were bred in house in the Biomedical and Behavioural Research Unit at University of Ulster, Coleraine. The original background and generation of these incretin receptor knockout mice are described elsewhere [39], [40]. GLP-1RKO and GIPRKO mice were backcrossed to wild type C57BL6/J mice for more than ten generations prior to use in the present study. Mice were housed individually in an air-conditioned room at 22 ± 2°C with a 12 h light and 12 h dark cycle. Standard rodent pellet diet (Trouw Nutrition, Northwich, Chesire, UK) and drinking water were available *ad libitum*. All animal experiments were carried out in accordance with the UK Animals (Scientific Procedures) Act 1986 and approved by the University of Ulster Animal Ethics Review Committee. All necessary steps were taken to ameliorate any potential animal suffering and animals were sacrificed by lethal inhalation of CO2 followed by cervical dislocation. For analysis of pregnancy induced changes, mice were culled at 18.5 day post coitum to procure pancreatic tissues, intestines and terminal blood samples (non-fasted) and compared to age matched non-pregnant control mice (n = 6 in each group). Glucose tolerance, insulin sensitivity, food intake and body weight changes were not monitored for fear of adverse effects on pregnancy outcomes.

### Immunohistochemistry

Pancreatic tissues and small intestines from non-pregnant and pregnant mice fixed in 4% paraformaldehyde for 48 h at 4 °C. The tissues were processed using automated tissue processor (Leica TP1020, Leica Microsystems, Nussloch, Germany). After embedding, tissues were sectioned at 7 µm using a microtome (Shandon finesse 325, Thermo scientific, UK). Six pancreatic sections were picked at an interval of 80 µm for histology. The tissue sections were deparaffinised and rehydrated through series of ethanol concentrations. After antigen retrieval at 94 °C for 20 min using citrate buffer (pH 6.0), the sections were blocked using 10% normal goat serum and incubated with primary antibody as appropriate: mouse monoclonal anti-insulin antibody (ab6995, 1∶1000; Abcam), gunieapig anti-glucagon antibody (PCA2/4, 1∶200; raised in-house), rabbit anti-GLP-1 antibody (XJIC8, 1∶200; raised in-house, specific for total GLP-1), rabbit anti-GIP antibody (RIC34/111J, 1∶400; kindly donated by Professor L Morgan, Guildford, UK), rabbit anti-Ki67 antibody (ab15580, 1∶200; Abcam), mouse anti-PC1/3 antibody (ab55543, 1∶100; Abcam) or rabbit anti-PC2 antibody (ab15610, 1∶200, Millipore), overnight at 4 °C. The sections were then incubated with secondary antibody (Alexa Fluor 488 goat anti-guinea pig IgG – 1∶400, Alexa Fluor 594 goat anti-mouse IgG – 1∶400, Alexa Fluor 488 goat anti-rabbit IgG – 1∶400 or Alexa Fluor 594 goat anti-rabbit IgG – 1∶400) as appropriate for 45 min at 37 °C. The slides were then mounted using anti-fade mounting medium and viewed under FITC filter (488 nm) or TRITC filter (594 nm) using fluorescent microscope (Olympus system microscope, model BX51) and photographed using the DP70 camera adapter system. Antibodies selected for immunohistochemical staining of glucagon, GLP-1 and GIP were highly specific and showed no cross reactivity with related peptide hormones. For analysis of beta cell apoptosis, Tunel assay was performed according to manufacturer's instructions (In situ cell death kit, Fluorescein, Roche Diagnostics, UK).

### Image analysis

Cell∧F image analysis software (Olympus Soft Imaging Solutions, GmbH) was used to analyse islet parameters including islet area, beta cell area and alpha cell area, expressed as µm^2^. The image folders were coded and the investigator was blinded during analysis of islet parameters. Number of islets per mm^2^ of pancreas and the proportion of islets with central alpha cells were determined in a blinded fashion. For analysis of islet size distribution, islets smaller than 10,000 µm^2^ were designated ‘small’, greater than 10,000 µm^2^ and less than 25,000 µm^2^ were designated ‘medium’ and greater than 25,000 µm^2^ were designated ‘large’. Colocalization analysis was carried out using ‘Colocalization finder’ plugin in ImageJ and expressed as Pearson's colocalization coefficient. A value close or equal to −1.0 represents no colocalization while a value close or equal to 1.0 represents full colocalization of two antigens. Alpha cells expressing GLP-1 or GIP with no visible glucagon staining were designated ‘GLP-1^+^/GIP^+^ glucagon^−^ cells. Ki67 and TUNEL positive, insulin positive cells were counted in a blinded manner and expressed as % of total number of beta cells analysed. Approximately 2000 beta cells per replicate were assessed. The balance between proliferation and apoptosis was expressed as ratio of Ki67/Tunel. For analysis of intestinal parameters including L cell count, K cell count and villus length, serial images of sections were photographed. Cell∧F image analysis software was used to determine mucosal area in mm^2^. Cell count was determined in a blinded manner and expressed as count per mm^2^ mucosal area. Villus length was determined using ‘line’ tool in Cell∧F software and expressed as µm.

### Biochemical analyses

Blood samples were collected in fluoride/heparin microcentrifuge tubes (Sarstedt, Numbrecht, Germany) and centrifuged for 30 s at 13,000 × g. Plasma was separated and stored at −80°C until analysis. Plasma glucose was measured by an automated glucose oxidase procedure (Beckman Glucose Analyzer). Pancreatic and intestinal tissues were extracted using buffer containing 20 mM Tris HCl (pH 7.5), 150 mM NaCl, 1 mM EDTA, 1 mM EGTA and 1% Triton × 100 and stored at −80 °C. Biochemical analyses were carried out for insulin by radioimmunoassay [42], total GLP-1 (GLP-1 total ELISA, EZGLP-1T-36K, Millipore), GIP (rat/mouse GIP ELISA, EZRMGIP-55K, Millipore) and glucagon (glucagon chemiluminescent assay, EZGLU-30K, Millipore) by specific enzyme linked immunoassays following the manufacturers' instructions. All commercial assay kits have been shown to exhibit a high degree of specificity.

### Statistics

Results were analysed in GraphPad PRISM (Version 5.0) and presented as mean ± SEM. Statistical analyses were carried out by unpaired Student's t test (non-parametric, with two-tailed P values and 95% confidence interval) and one way ANOVA with Bonferroni post-hoc test wherever applicable. Results were considered significant if p<0.05.

## Results

### Pregnancy significantly increases islet cell mass

**Figure 1:**
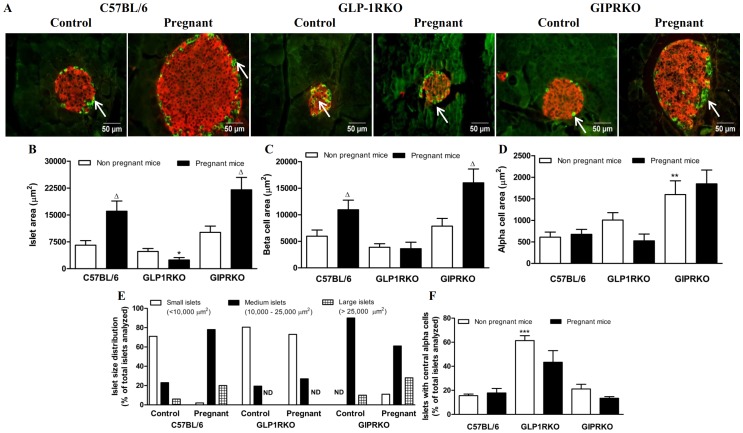
Islet analysis. A: Representative islets showing insulin (red) and glucagon (green, indicated by arrows) immunoreactivity from non-pregnant and pregnant C57BL/6, GLP-1RKO and GIPRKO mice are shown. B: Islet area, expressed as µm^2^. C: Beta cell area, expressed as µm^2^. D: Alpha cell area, expressed as µm^2^. E: Islet size distribution, expressed as % of total islets analysed (n  =  15 to 20 islets per animal). F: Islets with central alpha cells, expressed as percentage of total islets analysed (n  =  15 to 20 islets per animal). Values are mean ± SEM of 6 observations unless otherwise indicated. ^Δ^p<0.05 compared to respective non-pregnant controls. ^*^p<0.05, ^**^p<0.01, ^***^p<0.001 compared to non-pregnant or pregnant C57BL/6 mice. ND, none detected.

Representative islets depicting insulin/glucagon immunoreactivity from non-pregnant and pregnant C57BL/6, GLP-1RKO and GIPRKO mice are shown in Figure 1A. In C57BL/6 and GIPRKO animals, pregnancy significantly increased islet area and beta cell area by 1.8–2.5 fold and 1.4–2 fold respectively (p<0.05, Figure 1B, C). However in GLP-1RKO animals, pregnancy did not increase islet or beta cell area (Figure 1B, C) and quantification of islet area demonstrated a significant reduction in pregnant GLP-1RKO mice (p<0.05 vs. C57BL/6 mice, Figure 1B). Alpha cell area was unaffected by pregnancy in C57BL/6 and GIPRKO mice but a trend towards reduction in alpha cell area was observed in GLP-1RKO mice. Alpha cell area was significantly higher in non-pregnant and pregnant GIPRKO mice compared to C57BL/6 mice (p<0.01, Figure 1D).

**Figure 2:**
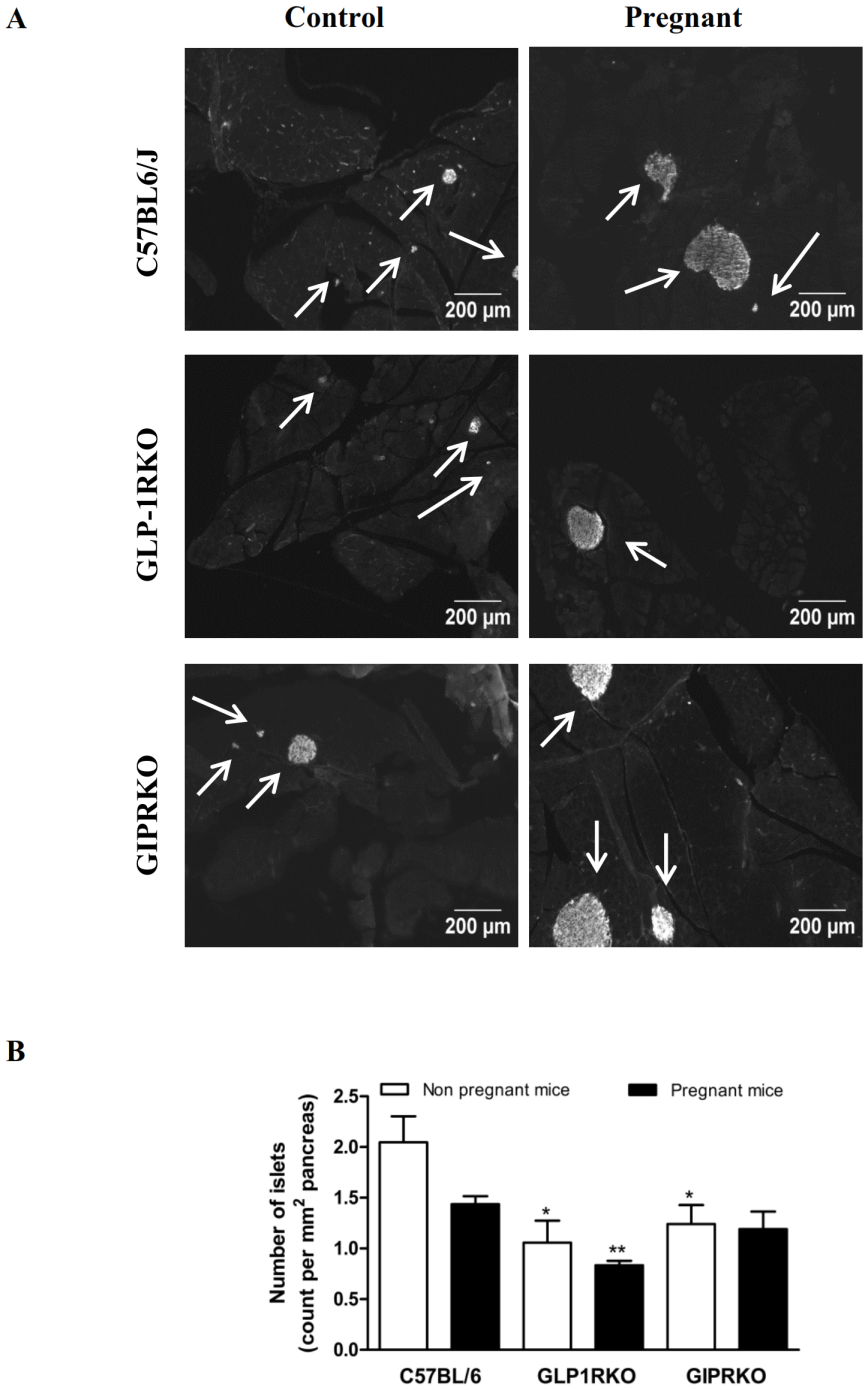
Islet analysis. A: Representative images showing islets from non-pregnant and pregnant C57BL/6, GLP-1RKO and GIPRKO mice are shown. B: Number of islets, expressed as count per mm^2^ of pancreas. Values are mean ± SEM of 6 observations unless otherwise indicated. ^*^p<0.05, ^**^p<0.01, ^***^p<0.001 compared to non-pregnant or pregnant C57BL/6 mice.

Pregnancy also affected islet size distribution, with percentage of medium and large sized islets increasing in C57BL/6 mice and percentage of large islets increasing in GIPRKO mice (Figure 1E). However in GLP-1RKO animals, pregnancy did not affect islet size distribution (Figure 1E). Defective GLP-1R signalling significantly affected islet topology, in that percentage of islets with centrally located alpha cells was markedly higher than non-pregnant wild type mice (p<0.001, Figure 1A, F). Representative images showing islet distribution in pancreas of non-pregnant and pregnant C57BL/6, GLP-1RKO and GIPRKO mice are shown in Figure 2A. Pregnancy did not affect number of islets per mm^2^ pancreas (Figure 2B). Non-pregnant receptor knockout mice demonstrated significantly lower number of islets compared to non-pregnant C57BL/6 mice (p<0.05, Figure 2B). Pregnant GLP-1RKO mice also had a lower number of islets compared to pregnant C57BL/6 mice (p<0.01, Figure 2B).

**Figure 3:**
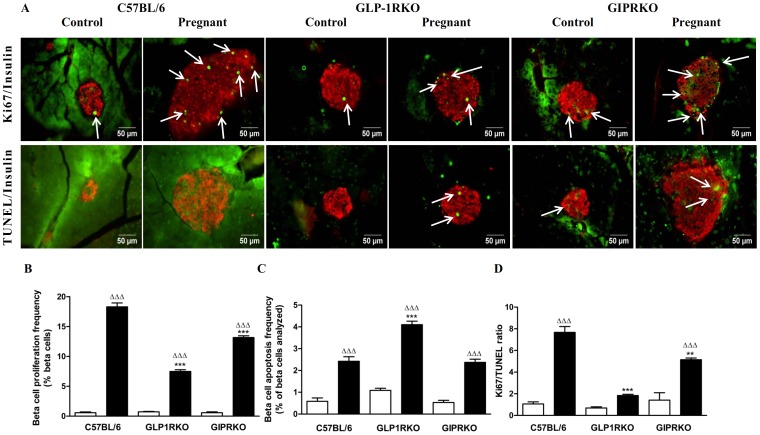
Proliferation and apoptosis frequencies. A: Representative islets showing insulin (red) and Ki67 (green, indicated by arrows), insulin (red) and TUNEL (green, indicated by arrows) immunoreactivity from non-pregnant and pregnant C57BL/6, GLP-1RKO and GIPRKO mice are shown. B: Beta cell proliferation frequency, expressed as % of beta cells analysed. C: Beta cell apoptosis frequency, expressed as % of beta cells analysed. D: Ki67/TUNEL ratio. Values are mean ± SEM of 6 observations unless otherwise indicated. ^ΔΔΔ^p<0.001 compared to respective non-pregnant controls. ^**^p<0.01, ^***^p<0.001 compared to non-pregnant or pregnant C57BL/6 mice.

Representative islets showing Ki67/insulin, TUNEL/insulin immuno-reactivity from non-pregnant and pregnant C57BL/6, GLP-1RKO and GIPRKO mice are shown in Figure 3A. Pregnancy significantly increased beta cell proliferation and apoptosis frequency in islet beta cells of all groups (p<0.001, Figure 3B, C). However, in pregnant GLP-1RKO and GIPRKO mice, beta cell proliferation frequency was markedly lower when compared with C57BL/6 mice (p<0.001, Figure 3B). The ratio of Ki67/TUNEL was higher in pregnant C57BL/6 and GIPRKO mice when compared with non-pregnant mice, thus favouring increased beta cell mass (p<0.05, Figure 3D). However in GLP-RKO mice, ratio of Ki67/TUNEL was not altered by pregnancy. The ratio of Ki67/TUNEL in pregnant GLP-1RKO and GIPRKO mice was significantly less than pregnant C57BL/6 mice (p<0.01, p<0.001, Figure 3D).

### Pregnancy alters proglucagon processing in alpha cells

**Figure 4:**
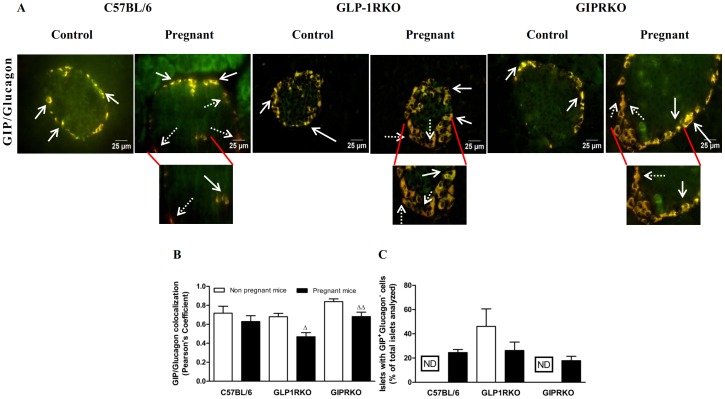
Glucagon and GIP colocalization. A: Representative islets showing glucagon (green) and GIP (red, indicated by block arrows) immunoreactivity from non-pregnant and pregnant C57BL/6, GLP-1RKO and GIPRKO mice are shown. Dotted arrows indicate alpha cells expressing mainly of GIP. B: GIP/glucagon colocalization, expressed in terms of Pearson's coefficient of colocalization. C: Islets with GIP^+^ glucagon^−^ cells, expressed as % of total islets analysed (n  =  15 to 20 islets per animal). Values are mean ± SEM of 6 observations unless otherwise indicated. ^Δ^p<0.05, ^ΔΔ^p<0.01 compared to respective non-pregnant controls. ND, not detected.

**Figure 5:**
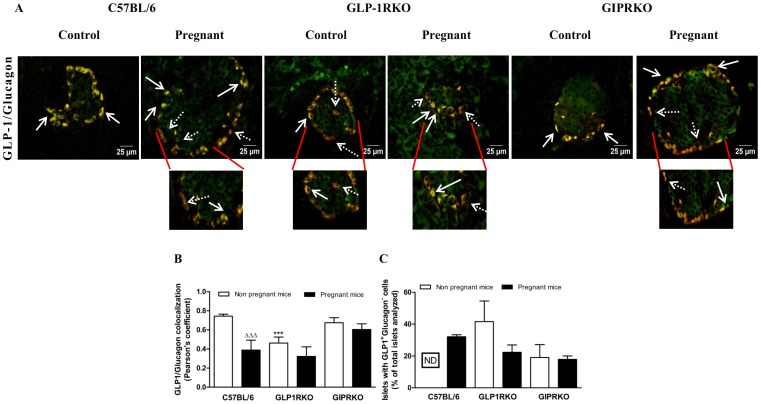
Glucagon and GLP-1 colocalization. A: Representative islets showing glucagon (green) and GLP-1 (red, indicated by block arrows) immunoreactivity from non-pregnant and pregnant C57BL/6, GLP-1RKO and GIPRKO mice are shown. Dotted arrows indicate alpha cells expressing mainly of GLP-1. B: GLP-1/glucagon colocalization, expressed in terms of Pearson's coefficient of colocalization. C: Islets with GLP-1^+^ glucagon^−^ cells, expressed as % of total islets analysed. Values are mean ± SEM of 6 observations. ^ΔΔΔ^p<0.001 compared to respective non-pregnant controls. ^***^p<0.001 compared to non-pregnant or pregnant C57BL/6 mice. ND, not detected.

Representative islets showing GIP/glucagon, GLP-1/glucagon colocalization from non-pregnant and pregnant C57BL/6, GLP-1RKO and GIPRKO mice are shown in Figures 4A and 5A respectively. Pregnancy did not affect GIP/glucagon colocalization in alpha cells in C57BL/6 mice (Figure 4B). GIP expression in both groups of receptor knockout mice was significantly lowered by pregnancy (p<0.05, p<0.01, Figure 4B). Interestingly, pregnancy lowered GLP-1/glucagon colocalization in alpha cells of C57BL/6 mice (p<0.001, Figure 5B). However this was primarily due to substantial increase in cells expressing solely GLP-1, indicated by dotted arrows pointing to GLP-1 immunoreactivity (red) (Figure 5A). Further evaluation revealed a substantial increase of glucagon deficient, GLP-1 positive or GIP positive cells in islets of pregnant C57BL/6 mice (Figures 4C and 5C). In contrast to both groups of KO mice, such cell populations were not observed in islets of non-pregnant C57BL/6 mice (Figures 4C and 5C). Pregnancy lowered GLP-1/glucagon colocalization in C57BL/6 mice (p<0.001, Figure 5B). GLP-1/glucagon colocalization in non-pregnant GLP-1RKO mice was significantly lower than non-pregnant C57BL/6 mice (p<0.001, Figure 5B), which was due to increased numbers of cells with predominant GLP-1 expression in GLP-1RKO mice (Figure 5C).

**Figure 6:**
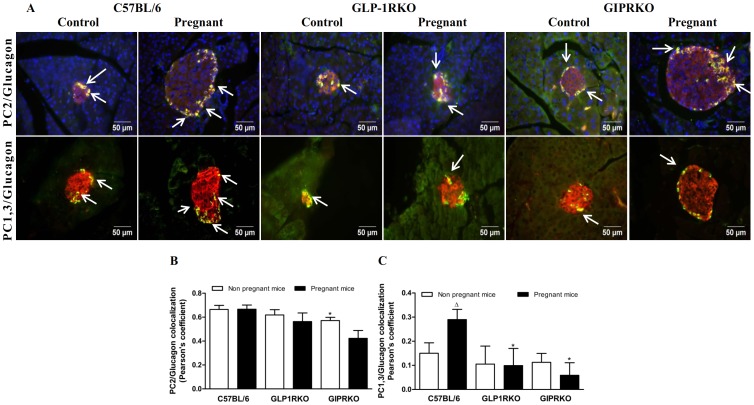
Glucagon and PC2 or PC1/3 colocalization. A: Representative islets showing glucagon (green) and PC2 (red, indicated by block arrows), glucagon (green) and PC1/3 (red, indicated by block arrows) immunoreactivity from non-pregnant and pregnant C57BL/6, GLP-1RKO and GIPRKO mice are shown. B: PC2/glucagon colocalization, expressed in terms of Pearson's coefficient of colocalization. C: PC1/3/glucagon colocalization, expressed in terms of Pearson's coefficient of colocalization. Values are mean ± SEM of 6 observations. ^Δ^p<0.05 compared to respective non-pregnant controls. ^*^p<0.05 compared to non-pregnant C57BL/6 mice.

Representative islets showing PC2/glucagon, PC1/3/glucagon immunoreactivity from non-pregnant and pregnant C57BL/6, GLP-1RKO and GIPRKO mice are shown in Figure 6A. PC2 and glucagon colocalization was not altered by pregnancy in any of the groups, despite strong tendency for reduction in GIPRKO mice (Figure 6B). Impairment of GIPR signalling reduced PC2/glucagon colocalization (p<0.05, Figure 6B). Pearson's colocalization coefficient for PC2/glucagon colocalization was approximately 0.7 while that for PC1/3/glucagon colocalization was approximately 0.2, clearly indicating that PC2 is the predominant prohormone convertase in alpha cells. Nevertheless, PC1/3 was detectable at low levels, as evidenced by positive values for colocalization coefficient (values closer to −1.0 represent no colocalization). PC1/3 levels in pregnant C57BL/6 mice were significantly increased (p<0.05, Figure 6C). PC1/3/glucagon colocalization in pregnant receptor knockout mice was significantly lower than pregnant C57BL/6 mice (p<0.05, Figure 6C).

### Pregnancy increases intestinal K/L cell count

**Figure 7:**
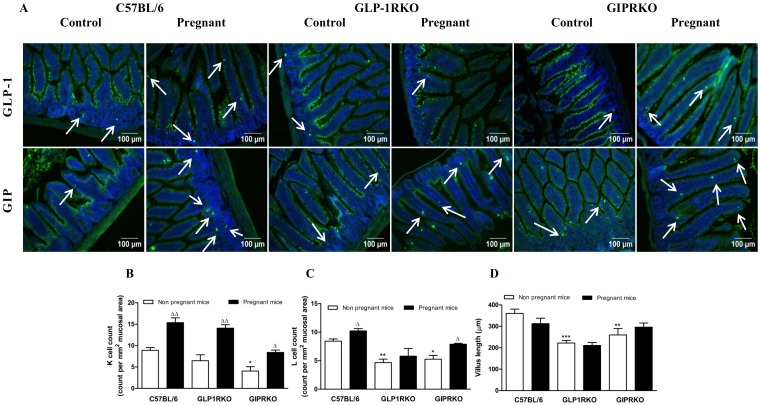
Intestine analyses. A: Representative images showing GLP-1 or GIP (green) positive cells in intestinal mucosa of non-pregnant and pregnant C57BL/6, GLP-1RKO and GIPRKO mice are shown. Arrows indicate GLP-1 or GIP positive cells. B: Intestinal K cell count, expressed as count per mm^2^ of mucosal area. C: Intestinal L cell count, expressed as count per mm^2^ of mucosal area. D: Villus length, expressed as µm. Values are mean ± SEM of 6 observations. ^Δ^p<0.05, ^ΔΔ^p<0.01 compared to respective non-pregnant controls. ^*^p<0.05, ^**^p<0.01, ^***^p<0.001 compared to non-pregnant or pregnant C57BL/6 mice.

Representative images showing GLP-1 or GIP positive cells in small intestinal mucosa of non-pregnant and pregnant C57BL/6, GLP-1RKO and GIPRKO mice are shown in Figure 7A. Pregnancy significantly increased intestinal K and L cell count in C57BL/6 and GIPRKO mice (p<0.05, p<0.01, Figure 7B, C). In GLP-1RKO mice, pregnancy increased K cell count but did not affect L cell count (p<0.01, Figure 7B, C). Defective receptor signalling decreased K and L cell count in GIPRKO mice and L cell count in GLP-1RKO mice compared to non-pregnant C57BL/6 mice (p<0.05, p<0.01, Figure 7B, C). Pregnancy did not affect villus length but defective receptor signalling significantly decreased villus length (p<0.01, p<0.001, Figure 7D).

### Pregnancy alters pancreatic, intestinal and plasma content of glucagon, GLP-1, GIP and insulin

**Figure 8:**
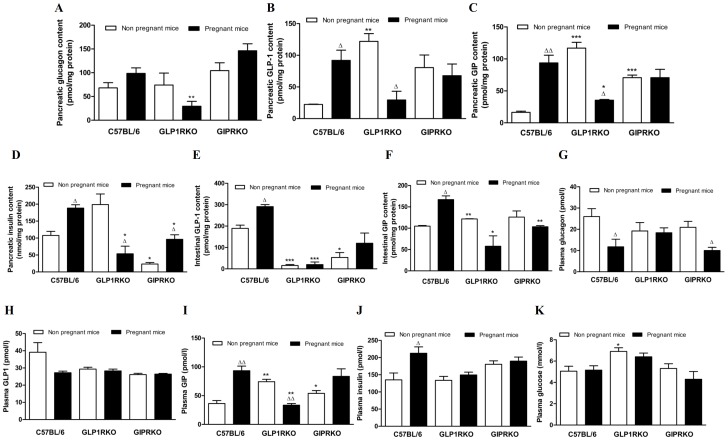
Pancreatic, intestinal and plasma levels of hormones. A: Pancreatic glucagon content, expressed as pmol/mg protein. B: Pancreatic GLP-1 content, expressed as pmol/mg protein. C: Pancreatic GIP content, expressed as pmol/mg protein. D: Pancreatic insulin content, expressed as nmol/mg protein. E: Intestinal GLP-1 content, expressed as pmol/mg protein. F: Intestinal GIP content, expressed as pmol/mg protein. G: Plasma glucagon, expressed as pmol/l. H: Plasma total GLP-1, expressed as pmol/l. I: Plasma GIP, expressed as pmol/l. J: Plasma insulin, expressed as pmol/l. K: Plasma glucose, expressed as mmol/l. Values are mean ± SEM of 4 observations unless otherwise indicated. ^Δ^p<0.05, ^ΔΔ^p<0.01 compared to respective non-pregnant controls. ^*^p<0.05, ^**^p<0.01, ^***^p<0.001 compared to non-pregnant or pregnant C57BL/6 mice.

Pregnancy did not affect pancreatic glucagon content in any of the groups but pancreatic glucagon content in pregnant GLP-1RKO mice was significantly lower than pregnant C57BL/6 mice (p<0.01, Figure 8A). Pregnancy markedly increased pancreatic GLP-1, GIP and insulin content in C57BL/6 mice (p<0.05, p<0.01, Figure 8B, C, D) and pancreatic insulin content in GIPRKO mice (p<0.05, Figure 8D). Defective GLP-1R signalling in non-pregnant mice markedly increased pancreatic GLP-1 and GIP content (p<0.01, p<0.001, Figure 8B, C) which was significantly reduced by pregnancy (p<0.05, Figure 8B, C). Pregnancy also decreased pancreatic insulin content in GLP-1RKO mice (p<0.05, Figure 8D). Defective GIPR signalling increased pancreatic GIP content (p<0.001, Figure 8C) which was unaffected in pregnancy. Pancreatic GIP and insulin content was significantly lower in pregnant GLP-1RKO mice when compared to pregnant C57BL/6 mice (p<0.05, Figure 8C, D).

Pregnancy markedly increased intestinal GLP-1 and GIP content in C57BL/6 mice but not in other groups (p<0.05, Figure 8E, F). Defective GLP-1 and GIP receptor signalling markedly reduced intestinal GLP-1 content (p<0.05, p<0.001, Figure 8E). Impairment of GLP-1 receptor signalling increased intestinal GIP content (p<0.01, Figure 8F), but levels were significantly decreased in pregnancy, compared with pregnant control C57BL/6 mice (p<0.05, Figure 8F). Intestinal GIP content was also reduced in pregnant GIPRKO mice when compared to pregnant C57BL/6 mice (p<0.01, Figure 8F).

In C57BL/6 mice, pregnancy significantly decreased plasma glucagon while it significantly increased plasma GIP and insulin, but not GLP-1 (p<0.05, p<0.01, Figure 8G, H, I, J). Pregnancy significantly decreased plasma GIP levels in GLP-1RKO mice (p<0.01, Figure 8I). Defective receptor signalling did not affect circulating levels of glucagon, total GLP-1 or insulin but increased levels of GIP (p<0.05, p<0.01, Figure 8I). Pregnancy did not alter plasma glucose in any of the groups although plasma glucose levels in non-pregnant GLP-1RKO mice were significantly higher than C57BL/6 mice (p<0.05, Figure 8K).

## Discussion

Pregnancy increases metabolic needs and hence alters maternal metabolism by enhancing nutrient absorption and pancreatic beta cell function to match increased demand. Earlier reports on pregnancy-induced beta cell compensation revealed that lactogenic hormones stimulate beta cell proliferation and suppress apoptosis, thereby increasing beta cell mass [36], [43]–[45]. This is supported by observations in prolactin receptor knockout mice which displayed an inability to increase beta cell mass during pregnancy [33], [45]. Several reports also claim that lactogenic hormones increase serotonin biosynthesis in beta cells which in turn exerts proliferative effects on beta cells [46], [47]. In contrast, the involvement of gut hormones which normally play a key role in the regulation of beta cell function and survival has been largely overlooked. Interestingly, Sugiyama et al. observed that beta cell proliferation was not affected in pregnant glucagon-GFP knock-in (Gcg^gfp^/Gcg^gfp^) mice lacking global proglucagon derived peptides, including glucagon, GLP-1, GLP-2 and oxyntomodulin [48]. However, recent studies have shown that these mice display important compensatory changes even in the non-pregnant state, including markedly increased circulating GIP with substantial ectopic expression of GIP in islet beta cells [29]. Thus, a positive role of GIP on beta cell mass may be particularly important in these animals. Accordingly, further studies are required using more specific receptor knockout models without functional GLP-1 or GIP receptors to assess the true role of incretin hormones in islet cell adaptations to pregnancy.

Our study using GLP-1R and GIPR knockout animal models revealed that receptor knockout did not affect islet area and beta cell area but increased alpha cell area without affecting pancreatic glucagon content in GIPRKO. As noted by Ling et al (2001), impairment of GLP-1R signalling did not affect pancreatic insulin or glucagon but increased the number of islets with centralised alpha cells [49]. Abolition of incretin signalling also decreased the number of islets in both groups of KO mice, possibly reflecting the impact of this pathway in postnatal neogenesis as suspected in transgenic mice overexpressing a dominant negative GIP receptor [50]. Recent studies have demonstrated the expression and secretion of GIP and GLP-1 in islets [20]–[26], [28]. Using specific antibodies, we consistently observed co-localisation of GIP, GLP-1, PC2 and PC1/3 together with glucagon in the alpha cells of normal as well as incretin receptor knockout mice. Further, appreciable amounts of GIP and GLP-1 were measured in pancreatic extracts, representing 11–15% of that present in the intestines. Knockout of GLP-1R resulted in compensatory increases in both pancreatic GIP and GLP-1, whereas abolition of GIPR increased pancreatic GIP. Interestingly, GLP-1R knockout mice exhibited a substantial increase in peripherally located islet cells which were either GLP-1 or GIP positive but glucagon deficient. GIPR KO mice similarly displayed substantial numbers of GLP-1 positive, glucagon deficient islet cells. The decrease of GLP-1/glucagon colocalization in alpha cells of GLP-1RKO and GIPRKO mice suggests that these incretin producing cells are derived from alpha cells. Indeed, there is evidence for a switch from PC2 to PC1/3 in islets exposed to increased functional demand or cytotoxic insult [20], [22], [24], [25]. Taken together, these observations in normal and knockout mice suggest that paracrine and other intra-islet effects of locally produced GIP and GLP-1 are likely to exert significant effects given their established actions on islet cell function [1]–[4], [41].

In contrast to the pancreas, few comprehensive observations have been made on the intestines of GLP-1R or GIPRKO mice. We observed that impairment of GLP-1R signalling decreased villus length, intestinal L cell count and GLP-1 content, with the expected compensatory increases of intestinal and circulating GIP [41], [51]. On the other hand, GIPRKO mice exhibited decreases in villus length, intestinal K and L cell counts and intestinal GLP-1 content, with paradoxical increase in circulating GIP. Others have reported normal or slightly elevated circulating GIP concentrations [52]. Thus, these data indicate that both GLP-1 and GIP are intimately involved in maintenance and function of incretin-producing enteroendocrine cell populations. It also appears that lack of functional GLP-1 is well compensated by enhancement of GIP, whereas compromised GIP action was not met with increases in circulating GLP-1.

As expected, pregnancy induced notable changes in the islet parameters of C57BL/6 mice, with increases in islet area, numbers of medium and large sized islets, beta cell area and both pancreatic and circulating insulin [33], [46], [48]. Pregnancy did not affect alpha cell area, pancreatic glucagon content or islet number, indicating that pregnancy was associated with expansion of beta cell mass rather than significant islet neogenesis as considered by others [53], [54]. GIP and GLP-1 were clearly expressed in islet alpha cells of pregnant mice and there was a substantial increase in peripherally located cell populations expressing only GIP or GLP-1. Consistent with these changes pancreatic levels of both GIP and GLP-1 were increased together with up-regulation of alpha cell PC1/3, as observed previously [22]. These changes were not accompanied by increases in GLP-1/glucagon colocalization but this could reflect a suspected increase of alpha cells expressing only GLP-1, hence giving a lower value for the colocalization coefficient. Similarly pregnancy increased intestinal contents of both incretin hormones and the numbers of intestinal K- and L-cells, resulting in increased circulating levels of GIP but notably no change in GLP-1. Consistent with negative intra-islet regulation by increased beta cell numbers and insulin content [55]–[57], plasma glucagon levels were decreased by pregnancy.

The changes considered above indicate significant adaptation of islets in pregnancy and suggest that they may be partly due to intra-islet production of GLP-1 and GIP which exerts local effects on beta cell function, including stimulation of beta cell proliferation and inhibition of apoptosis. The observed increase of circulating GIP suggests possible involvement of K-cell derived GIP but contrary to such a view, the pregnancy-induced changes in the islets of GIPR KO mice were similar to pregnant C57BL/6 mice. This included prominent increases in islet area, beta cell area and pancreatic insulin content together with decreased islet GIP/glucagon colocalization and no increase in the population of cells solely expressing GLP-1. Thus it appears that intestinal or islet derived GIP plays little role in islet adaptation to pregnancy. However, in marked contrast, pregnant GLP-1R KO mice exhibited no adaptive changes in any of these islet parameters and displayed an actual decrease of islet numbers. Consistent with decreased beta cell mass, the ratio of Ki67/TUNEL positive insulin positive cells in pregnant GLP-1R KO mice did not favour proliferation. Since circulating GLP-1 was unchanged in these animals, loss of islet compensation is more likely to be due to abolition of the intra-islet effects of islet-derived GLP-1. Furthermore in GLP-1RKO mice, pregnancy was not associated with increases in populations of islet cells purely expressing GLP-1 or GIP and there was a decrease in numbers of alpha cells co-expressing GIP and glucagon. Pancreatic GLP-1, GIP and insulin were actually decreased and the pregnancy-induced increases in intestinal GLP-1 and GIP together with L-cell hyperplasia were abolished. Taken together, these data reveal an essential role of GLP-1 in islet compensation to pregnancy, suggesting crucial involvement of changes in proglucagon processing in islet cells leading to local generation of GLP-1. Clearly generation of transgenic mice with targeted knockout of beta cell GLP-1 receptor could be useful to separate out any indirect effects.

The mechanisms responsible for effects of GLP-1 on beta cell mass clearly involve stimulation of beta cell proliferation and inhibition of apoptosis. Evidence suggests that this is regulated by PI3K signalling pathway [58]–[62]. Clearly other factors such as estrogens, prolactin, serotonin and placental hormones may feed into the signalling pathways including involvement of PI3K for estrogens and prolactin. Further, although evidence is lacking, we cannot rule out possible effects of GLP-1RKO on secretion of pituitary hormones such as prolactin. Future research to understand the expansion of beta cell mass in human islets and the underlying involvement of GLP-1 activated pathways may well open up new avenues to enhance beta cell mass for the treatment of diabetes.
